# HOW CAN IMPROVING EMPLOYMENT PROSPECTS OF UNEMPLOYED OLDER WORKERS BE EXPLAINED?

**DOI:** 10.1093/geroni/igz038.2806

**Published:** 2019-11-08

**Authors:** Per H Jensen

**Affiliations:** Center for Comparative Welfare Studies, Aalborg University, Nordjylland, Denmark

## Abstract

Until recently employment prospects of older workers have been rather poor. In recent years, however, the duration of unemployment among older workers has diminished, meaning that older workers are more frequently hired by employers. Changing employment prospects of older workers are no doubt framed by a decrease in overall unemployment. The aim of this paper, however, is to shed light on the emergence of new inclusive mechanisms by answering three interrelated research questions: (1) where are the job openings for unemployed seniors? (2) How have unemployed seniors been recruited? (3) Why do companies hire older workers. Using Denmark as a test case findings show that job openings are rather frequent in branches with tight labor markets and that characteristics of companies and management are important; for instance, the older the average age of management the higher the inclination to hire unemployed older workers. Findings furthermore show that mouth-to-mouth recommendation and the internet are the most used recruitment channels, while the public employment service is less used. Finally, findings show that qualification, stability and experience are the most reported reasons as to why employers hire unemployed older workers are. The paper is based on a survey with 2,525 valid respondents, response rate: 25.

